# Demographic and clinical characteristics of hospitalized children with suspected and laboratory-confirmed COVID-19 during the six waves of SARS-CoV-2 infection in northern Iran a retrospective cohort study

**DOI:** 10.3389/fped.2024.1288448

**Published:** 2024-12-05

**Authors:** Mohsen Mohammadi, Erfan Hashemi, Hoda Shirafkan, Farzin Sadeghi, Yousef Yahyapour

**Affiliations:** ^1^ Non-Communicable Pediatric Disease Research Center, Health Research Institute, Babol University of Medical Sciences, Babol, Iran; ^2^ Student Research Committee, Babol University of Medical Sciences, Babol, Iran; ^3^ Social Determinants of Health Research Center, Health Research Institute, Babol University of Medical Science, Babol, Iran; ^4^ Cellular and Molecular Biology Research Center, Health Research Institute, Babol University of Medical Sciences, Babol, Iran; ^5^ Department of Medical Microbiology and Biotechnology, Faculty of Medicine, Babol University of Medical Sciences, Babol, Iran; ^6^ Infectious Diseases and Tropical Medicine Research Center, Health Research Institute, Babol University of Medical Sciences, Babol, Iran

**Keywords:** COVID-19, SARS-CoV-2, hospitalized, children, outcome, ICU

## Abstract

**Introduction:**

Northern Iran is one of the regions of the country most affected by COVID-19. The aim of the current study was to examine the demographics, clinical characteristics, and outcomes of suspected and laboratory-confirmed patients <18 years of age hospitalized over two years (during six waves of SARS-CoV-2).

**Methods:**

This retrospective cohort study examined hospitalized patients with suspected SARS-CoV-2 infection in Babol, northern Iran. The study included patients <18 years of age admitted to a pediatric referral hospital from March 7, 2020 to March 20, 2022. Epidemiological and demographic characteristics, real-time PCR cycle threshold (Ct), clinical data and COVID-19 results were analyzed in the hospitalized patients.

**Results:**

Totally, 2,731 patients with suspected COVID-19 were examined from March 2020 to 2022, with a mean age of 4.6 years, and were male (54.3%). Most suspected cases occurred during the fifth (Delta) and sixth (Omicron) relapse (30% and 20%, respectively). Among them, 461 patients had comorbidities, with brain and neurological diseases (BND), malignancies and hematopoietic and blood disorders (HBDs) being the most common (6.7%, 2.6% and 2.1%, respectively). Real-time RT- PCR showed that 391 patients were positive. The mean age of the positive patients was 5.2 years. Most positive cases were in the fifth wave (30.9%), male (53.7%) and 1–5 years old (49.1%). BND (9.5%) and cardiovascular diseases (CVD) (4.3%) were common underlying conditions. A higher viral load (Ct score = 9–20) was most common in the fifth wave. Moreover, 8.7% of ICU admissions and 2.4% of deaths were due to a Ct score of 9–20. Most deaths occurred in the 1–5 age group and were associated with BND. More deaths occurred in women (73.9% overall, 71.4% real-time RT- PCR -positive), and the highest mortality was in the fifth wave. The risk ratio of mortality was higher in children with kidney disease (KD) and BND.

**Conclusion:**

There were 27 ICU admissions (6.9%) and seven deaths (1.8%). The current study also revealed that children of any age can be susceptible to SARS-CoV-2 infection, while children aged 1–5 years are more susceptible to COVID-19. The Delta variant is associated with higher rates of hospitalization and ICU admission, and the presence of BND is associated with higher mortality rates.

## 1 Introduction

The pandemic caused by the severe acute respiratory syndrome coronavirus 2 (SARS-CoV-2) that led to coronavirus disease 2019 (COVID-19) is a global health crisis of unprecedented magnitude in the twenty-first century (1). According to information from the World Health Organization, as of August 2023, more than 769 million cases have been confirmed, and approximately 7 million deaths have been reported worldwide (2, 3).

The pandemic has highlighted the importance of rapid and ongoing evaluation of the SARS-CoV-2 spreading patterns (2). To determine the appropriate course of action for public health, it is essential to fully comprehend the transmission, pathogenicity, and clinical alterations associated with certain variations (4–6). However, initial investigations have primarily focused on adults and the elderly because of the symptoms-based SARS-CoV-2 diagnosis at the initiation of the pandemic and due to the mild or asymptomatic nature of the SARS-CoV-2 presentation among most children resulted in screening strategies evasion, under diagnosis, and garnering less attention for this population (7, 8); Therefore, due to a lack of adequate investigations of SARS-CoV-2 in children, our knowledge of the virus's prevalence and pattern among children in different surges was limited. Recently, attention has been garnered due to recent reports about the increase in the transmission of specific variants of SARS-CoV-2, such as the Delta variant, in all age groups, including children (9, 10).

Studies observed that the COVID-19 virus typically presents with mild symptoms or may even be asymptomatic in children, with a relatively low mortality rate (11, 12); However, COVID-19 can not only affect the mental health of this group of patients and result in psychological disorders (13), but it can also be associated with life-threatening conditions, which cannot be neglectable, including pediatric inflammatory syndrome with multisystem involvement (MIS-C), Septic shock, toxic encephalopathy, multiple organ failure syndromes, and disseminated intravascular coagulation (DIC), especially in patients with an underlying medical condition (13–16).

Although recently, the different surges of the SARS-CoV-2 pandemic have been the focus of a vast amount of investigations, few studies have compared the demographic properties, manifestations, and complications of each SARS-CoV-2 pandemic surge, especially in hospitalized children of different age groups compared to adults (17), even for the COVID-19 vaccination side effects (18, 19). Moreover, based on our knowledge, there is a remarkable lack of studies evaluating hospitalized children's characterizations during the 6 SARS-CoV-2 waves, especially in the Middle East. Therefore, the current study aimed to fill the gaps and better understanding of the patterns and characterization of the different COVID-19 surges among children by providing a demographic and clinical characterization.

## 2 Methods

### 2.1 Study design and setting

A retrospective cohort study was conducted with the support of the Institutional Ethics Committee of Babol University of Medical Sciences (IR.MUBABOL.HRI.REC.1401.271) in two medical research and teaching hospitals, Amirkola and Rohani Hospitals in Babol, Iran. All hospitalized children with suspected or laboratory-confirmed SARS-CoV-2 infection from March 7, 2020 to March 20, 2022 were entered into this retrospective multicenter cohort study.

It is necessary to mention that all hospitalized children with suspected or laboratory-confirmed SARS-CoV-2 infection were sampled from our previous study (1). All hospitalized patients were followed up until discharge or death. The inclusion criteria were hospitalizations with a suspected diagnosis of COVID-19 during the study.

Subjects without a real-time polymerase chain reaction (RT-PCR) test result and duplicate records based on name, ID number and gender were excluded from the study.

### 2.2 Data collection and sources

The study population was patients <18 years of age suspected of having COVID-19. The criteria for defining suspected COVID-19 cases were those of the WHO (20). In pediatric hospitals, respiratory or inflammatory symptoms were considered suspected cases of COVID-19.

SARS-CoV-2 infection was confirmed by RT-PCR at the SARS-CoV-2 Molecular Diagnostic Reference Laboratory affiliated with Babol University of Medical Sciences. For each patient, demographic variables such as age and gender, self-reported history of underlying diseases—including cardiovascular disease (CVD), diabetes, hypertension, kidney disease (KD), cancer, brain and neurologic disorders (BND), RT-PCR result, ICU admission and hospital outcome (discharge or death) were recorded.

### 2.3 Real-time PCR (RT-PCR) and relative viral load

Extraction of viral nucleic acid and RT-PCR for the detection of SARS-CoV-2 and determination of variants of concern (VOCs) were performed as explained in the previous study (17). Briefly, the current study utilized the GA SARS-CoV-2 One-Step RT-PCR Kit (GeneovA, Iran), a commercial triple-target assay developed for the detection of specific mutations in the S, N, and ORF1a genes of SARS-CoV-2. According to a recently published study, there is good agreement between the GA SARS-CoV-2 One-Step RT-PCR Kit and NGS for the identification of SARS-CoV-2 variants of concern (VOCs) (21). By the cycle threshold (Ct) values obtained from the RT-PCR, the viral loads of the SARS-CoV-2 positive samples were relatively determined. The CT cutoff value of this kit was 40 and samples with a Ct value ≤40 were positive. In the present study, Ct values were used to determine SARS-CoV-2 viral load as copies/ml. It was calculated using the following equation *y* = −3.2733 × +38.59, as described in a previous study, and classified into three groups: with Ct values between 9 and 20 (3.28 × 10^11^ - 1.43 × 10^8^ copies/ml), 21–30 (7.09 × 10^7^ - 1.26 × 10^5^ copies/ml), and 31–40 (6.25 × 10^4^ - 1.11 × 10^2^ copies/ml) (22).

### 2.4 COVID-19 waves

Within two years, from March 7, 2020 to March 20, 2022, there were six waves of COVID-19 in Mazandaran province and Babol district. The first wave started on March 7, 2020 and ended on May 16, 2020 (approximately 71 days); the second wave started on June 10, 2020 and ended on September 5, 2020 (approximately 88 days); the third wave started on November 25, 2020 and ended on March 5, 2021 (approximately 101 days); the fourth wave started on March 25, 2021 and ended on May 31, 2021 (approximately 68 days, with Alpha variant being the dominant circulating variant); the fifth wave started on June 26, 2021 and ended on December 1, 2021 (approximately 159 days, with the Delta variant being the dominant circulating variant). The sixth wave started on January 17, 2022 and lasted until March 20, 2022 (approximately 63 days, with the Omicron variant being the dominant circulating variant). In the first three waves of COVID-19, the ancestral lineage of SARS-CoV-2 was the dominant circulating variant. In addition, there were about 208 days between waves (as a null wave) in two years.

### 2.5 Statistical analysis

The data were analyzed using SPSS 22. Categorical variables are summarized as frequencies and percentages. Quantitative variables were presented as mean ± standard deviation (SD), median and IQR. The Pearson chi-square test, the generalized Fisher exact test, the Cochrane-Armitage test for trends, the t test for independent samples and the Mann-Whitney U test were used to compare groups or categories. Moreover, the median and Kruskal-Wallis test were used to compare the median and mean of ages between groups. Additionally, to assess the factors influencing patient survival, a multivariable Cox regression was also performed. In the first step, a univariate Cox regression was done. Age, gender, mean Ct and underlying diseases were entered into the model as independent parameters. In the second step, all variables were included in the multivariable Cox regression. The coefficients were estimated using the Wald method. A value of *p* < 0.05 was considered significant.

## 3 Result

### 3.1 Characteristics of patients with suspected COVID-19

From March 2020 to 2022, 2,731 suspected SARS-CoV-2 infected patients aged <18 years with a mean age of 4.6 ± 4.6 years (median: 3, IQR: 6) were enrolled in the current study (56.2% = 1–5 years, 54.3% = male).

Regardless of the null wave, most suspected cases of COVID-19 belong to the fifth (Delta) and sixth (Omicron) waves of SARS-CoV-2, with an incidence of 30% and 20% among the waves, respectively. Among them, 461 had at least one comorbidity. BND, malignancies and hematopoietic and blood disorders (HBDs) were the most common underlying conditions at 6.7%, 2.6% and 2.1%, respectively. The median length of hospitalization was five days, with an interquartile range of 4 days. Of the total hospitalized patients, 85 were admitted to the ICU (Table 1).

**Table 1:** Characteristics and outcomes in 2,731 hospitalized children during the period when SARS-CoV-2 variant was prevalent in Babol district, northern Iran in six waves (From March 7, 2020 to March 20, 2022).

Variable	Total *N* (%)	First (Ancestral SARS-CoV-2) wave *N* (%)	Second (Ancestral SARS-CoV-2) wave *N* (%)	Third (Ancestral SARS-CoV-2) wave *N* (%)	Fourth (Alpha) wave *N* (%)	Fifth (Delta) wave *N* (%)	Sixth (Omicron) wave *N* (%)	Null wave* N (%)	*p*-value
Overall	2,731	89	270	304	185	522	349	1,012	–
Median age, yrs, (IQR)	3 (6)	2 (9)	3 (7)	3 (6.75)	3 (6)	3 (6)	2 (4)	3 (6)	0.145^a^
Mean ± SD, age, yrs	4.6 ± 4.6	5 ± 8.8	5.1 ± 5.1	4.6 ± 4.5	4.8 ± 4.5	4.7 ± 4.6	3.8 ± 3.8	4.6 ± 4.5	0.057^b^
Age group, yrs									
<1	313 (11.5)	37 (41.6)	51 (18.9)	26 (8.6)	14 (7.6)	40 (7.7)	28 (8)	117 (11.6)	<0.001^c^
1–5	1,535 (56.2)	16 (18)	127 (47)	175 (57.6)	107 (57.8)	312 (59.8)	235 (67.3)	563 (55.6)	
6–10	514 (18.8)	16 (18)	45 (16.7)	63 (20.7)	42 (22.7)	97 (18.6)	58 (16.6)	193 (19.1)	
11–18	369 (13.5)	20 (22.5)	47 (17.4)	40 (13.2)	22 (11.9)	73 (14)	28 (8)	139 (13.7)	
Gender									
Boy	1,483 (54.3)	48 (53.9)	154 (57)	179 (58.9)	92 (49.7)	269 (51.1)	184 (52.7)	557 (55)	0.309^c^
Girl	1,248 (45.7)	41 (46.1)	116 (43)	125 (41.1)	93 (80.3)	253 (48.5)	165 (47.3)	445 (45)	
Real-time PCR results									
Positive	391 (14.3)	3 (3.4)	70 (25.9)	30 (9.9)	18 (9.7)	121 (23.2)	84 (24.1)	65 (6.4)	<0.001^c^
Negative	2,340 (85.7)	86 (96.6)	200 (74.1)	274 (90.1)	167 (90.3)	401 (76.8)	265 (75.9)	947 (93.6)	
Underlying diseases									
CVD^1^	51 (1.9)	3 (3.4)	7 (2.6)	3 (1)	3 (1.6)	12 (2.3)	6 (1.7)	17 (1.7)	0.679^c^
Diabetes	24 (0.9)	3 (3.4)	1 (0.4)	3 (1)	2 (1.1)	4 (0.8)	2 (0.6)	9 (0.9)	0.259^c^
KD^2^	52 (1.9)	1 (1.1)	8 (3)	13 (4.3)	1 (0.5)	5 (1)	7 (2)	17 (1.7)	0.015^c^
Hypertension	6 (0.2)	0 (0.0)	1 (0.4)	1 (0.3)	0 (0.0)	0 (0.0)	0 (0.0)	4 (0.4)	0.636^c^
Malignancies	72 (2.6)	0 (0.0)	14 (5.2)	12 (3.9)	8 (4.3)	11 (2.1)	6 (1.7)	21 (2.1)	0.012^c^
BND^3^	183 (6.7)	2 (2.2)	24 (8.9)	14 (4.6)	14 (7.6)	33 (6.3)	17 (4.9)	79 (7.8)	0.082^c^
RD^4^	20 (0.7)	2 (2.2)	4 (1.5)	3 (1)	0 (0.0)	3 (0.6)	1 (0.3)	7 (0.7)	0.239^d^
GID^5^	5 (0.2)	0 (0.0)	2 (0.7)	3 (1)	0 (0.0)	0 (0.0)	0 (0.0)	0 (0.0)	0.006^d^
LD^6^	12 (0.4)	1 (1.1)	1 (0.4)	1 (0.3)	0 (0.0)	3 (0.6)	1 (0.3)	5 (0.5)	0.187^d^
HBD^7^	58 (2.1)	6 (6.7)	16 (5.9)	5 (1.6)	3 (1.6)	3 (0.6)	5 (1.4)	20 (2)	<0.001^c^
Others^8^	29 (1.1)	0 (0.0)	9 (3.3)	3 (1)	3 (1.6)	4 (0.8)	0 (0.0)	10 (1)	0.009^c^
Comorbidities									
No Comorbidities	2,270 (83.1)	73 (82)	201 (74.4)	250 (82.2)	153 (82.7)	448 (85.8)	305 (87.4)	840 (83)	<0.001^c^
One	411 (15)	15 (16.9)	53 (19.6)	47 (15.5)	28 (15.1)	70 (13.4)	43 (12.3)	155 (15.3)	
≥Two	50 (1.8)	1 (1.1)	16 (5.9)	7 (2.3)	4 (2.2)	4 (0.8)	1 (0.3)	17 (1.7)	
Hospitalization outcome									
Length of stay, days, median (IQR)	5 (4)	6 (5.5)	6 (4)	5 (4)	5 (4)	5 (4)	5 (4)	5 (3)	<0.001^c^
Length of stay, days, Mean ± SD	6.5 ± 6.4	8.7 ± 9.5	7.1 ± 6.3	7.2 ± 8	6 ± 4.9	6.5 ± 6.4	5.8 ± 4.6	6.3 ± 6	<0.001^b^
ICU admission	85 (3.1)	4 (4.5)	15 (5.6)	8 (2.6)	2 (1.1)	24 (4.6)	10 (2.9)	22 (2.2)	0.017^d^
In-hospital death	23 (0.8)	2 (2.2)	2 (0.7)	1 (0.3)	4 (2.2)	9 (1.7)	2 (1.6)	3 (0.3)	0.009^d^

1. CVD: cardiovascular diseases; 2. KD: kidney diseases; 3. BND: brain & neurologic disorders; 4. RD: respiratory diseases; 5. GID: gastrointestinal diseases; 6. LD: liver diseases; 7. HBD: hematopoietic & blood disorders; 8. others: including immunodeficiency diseases, special diseases, and thyroiditis.

^a^ Median test was used.

^b^ Kruskal-Wallis test was used for mean age groups.

^c^ Cochrane-Armitage test for linear associations.

^d^ Generalized Fisher exact test was used to compare groups.

* Null wave: referring to the time between waves of SARS-CoV-2 infections (approximately 208 days).

### 3.2 Characteristics of laboratory-confirmed patients (RT-PCR -positive)

Of all suspected cases, RT-PCR was positive in 391 patients. The mean age of the overall positive RT-PCR patients was 5.2 ± 5.3 years (median: 3, IQR: 8). Of all waves of SARS-CoV-2 infection, the highest number of RT-PCR-positive patients was recorded in the fifth wave with 121 cases (30.9%). Most RT-PCR-positive patients were male (53.7%) and 1–5 years old (49.1%). Except for the first wave, the age range of 1–5 years accounted for the largest number of patients in all waves of COVID-19 and even in the intervals between waves. BND (9.5%) and CVD (4.3%) were the two most common underlying diseases in RT-PCR-positive patients (Table 2).

**Table 2:** Characteristics and outcomes in 391 hospitalized patients with confirmed COVID-19 by real-time PCR in the period of prevalence of SARS-CoV-2 variant predominance in Babol district, northern Iran in six waves (From March 7, 2020 to March 20, 2022).

Variable	Total *N* (%)	First (Ancestral SARS-CoV-2) wave *N* (%)	Second (Ancestral SARS-CoV-2) wave *N* (%)	Third (Ancestral SARS-CoV-2) wave *N* (%)	Fourth (Alpha) wave *N* (%)	Fifth (Delta) wave *N* (%)	Sixth (Omicron) wave *N* (%)	Null wave* N (%)	*p*-value
Overall	391	3	70	30	18	121	84	65	–
Median age, yrs, (IQR)	3 (8)	12	3 (8)	1.5 (8.5)	5 (13)	2 (9)	2 (5.75)	3 (9.5)	0.145^a^
Mean ± SD, age, yrs	5.2 ± 5.3	10.3 ± 5.7	5.1 ± 5.5	4.9 ± 5.9	7.4 ± 6	5.4 ± 5.4	4.1 ± 4.3	5.8 ± 5.6	0.129^b^
Age group, yrs									
>1	52 (13.3)	0 (0.0)	16 (22.9)	5 (16.7)	0 (0.0)	15 (12.7)	8 (9.8)	8 (12.3)	0.076^c^
1–5	192 (49.1)	1 (33.3)	31 (44.3)	16 (53.3)	10 (55.6)	53 (43.8)	51 (60.7)	30 (46.2)	
6–10	70 (17.9)	0 (0.0)	10 (14.3)	2 (6.7)	3 (16.7)	27 (22.3)	17 (20.2)	11 (16.9)	
11–18	77 (19.7)	2 (66.7)	13 (18.6)	7 (23.3)	5 (27.8)	26 (21.5)	8 (9.5)	16 (24.6)	
Gender									
Boy	210 (53.7)	1 (33.3)	43 (61.4)	17 (56.7)	12 (66.7)	58 (47.9)	44 (52.4)	35 (53.8)	0.521^c^
Girl	181 (46.3)	2 (66.7)	27 (38.6)	13 (43.3)	6 (33.3)	63 (52.1)	40 (47.6)	30 (46.3)	
Mean Ct (Relative viral load)**									
9–20	126 (32.3)	0 (0.0)	12 (17.1)	8 (26.7)	4 (22.2)	55 (45.8)	32 (38.1)	15 (23.1)	<0.001^c^
21–30	143 (36.7)	0 (0.0)	19 (27.1)	14 (46.7)	8 (44.4)	40 (33.3)	43 (51.2)	19 (29.2)	
31–40	121 (31)	3 (100)	39 (55.7)	8 (26.7)	6 (33.3)	25 (20.8)	9 (10.7)	31 (47.7)	
Underlying diseases									
CVD^1^	17 (4.3)	0 (0.0)	2 (2.9)	0 (0.0)	2 (11.1)	7 (5.8)	2 (2.4)	4 (6.2)	–
Diabetics	5 (1.3)	0 (0.0)	0 (0.0)	1 (3.3)	0 (0.0)	2 (1.7)	1 (1.2)	1 (1.5)	–
KD^2^	11 (2.8)	0 (0.0)	3 (4.3)	0 (0.0)	0 (0.0)	1 (0.8)	5 (6)	2 (3.1)	–
Malignancies	7 (1.8)	0 (0.0)	2 (2.9)	0 (0.0)	1 (5.6)	1 (0.8)	3 (3.6)	0 (0.0)	–
BND^3^	37 (9.5)	0 (0.0)	8 (11.4)	2 (6.7)	3 (16.7)	9 (7.4)	7 (8.3)	8 (12.3)	–
HBD^4^	5 (1.3)	0 (0.0)	1 (1.4)	0 (0.0)	1 (5.6)	1 (0.8)	0 (0.0)	2 (3.1)	–
Others^5^	11 (2.8)	1 (33.3)	5 (7.1)	1 (3.3)	0 (0.0)	0 (0.0)	1 (1.2)	3 (4.6)	–
Comorbidities									
No comorbidities	306 (78.3)	2 (66.7)	52 (74.3)	26 (86.7)	12 (66.7)	102 (84.3)	66 (78.6)	40 (70.8)	0.492^c^
One	77 (19.7)	1 (33.3)	15 (21.4)	4 (13.3)	5 (27.8)	17 (14)	17 (20.2)	18 (27.7)	
≥Two	8 (2)	0 (0.0)	3 (4.3)	0 (0.0)	1 (5.6)	2 (1.7)	1 (1.2)	1 (1.5)	
Hospitalization outcome									
Length of stay, days, median (IQR)	4.5 (4)	6 (-)	6 (5.25)	4 (3)	4 (6.25)	4 (4)	5 (3)	4 (5)	0.116^c^
Length of stay, days, Mean ± SD	6.3 ± 6.6	7.7 ± 4.7	8.8 ± 9.8	4.9 ± 2.8	6.4 ± 4.8	5.5 ± 5.8	5.5 ± 5.5	6.8 ± 6	0.007^b^
ICU admission	27 (6.9)	0 (0.0)	5 (7.1)	3 (10)	0 (0.0)	12 (9.9)	6 (4.8)	3 (4.6)	0.624^d^
In-hospital death	7 (1.8)	0 (0.0)	1 (1.4)	1 (3.3)	0 (0.0)	3 (2.5)	2 (2.4)	0 (0.0)	0.817^d^

1. CVD: cardiovascular diseases; 2. KD: kidney diseases; 3. BND: brain & neurologic disorders; 4. HBD: hematopoietic & blood disorders; 5. others: including hypertension, respiratory diseases, gastrointestinal diseases, special diseases, thyroiditis, and immunodeficiency diseases.

^a^ Median test was used.

^b^ Kruskal-Wallis test was used for mean age groups.

^c^ Cochrane-Armitage test for linear associations.

^d^ Generalized Fisher exact test was used to compare groups.

* Null wave: referring to the time between waves of SARS-CoV-2 infections (approximately 208 days).

** The Ct value of a positive case was unknown.

### 3.3 SARS-CoV-2 relative viral load and outcome

Of the RT-PCR-positive patients, 126, 143, and 121 patients had a mean Ct of rRT-PCR in the ranges of 9–20, 21–30, and 31–40, respectively. Most patients with the highest relative viral load (Ct = 9–20) were detected in the fifth wave (related to the Delta variant).

Of 27 patients admitted to the ICU in the RT-PCR-positive group, 11 (40.7%) had a Ct value in the range of 9–20. The rate of ICU admission for patients with a Ct score of 9–20, 21–30 and 31–40 was 8.7% (11/126), 6.3% (9/143) and 5.8% (7/121), respectively. In addition, the rate of in-hospital mortality in patients with a Ct score of 9–20, 21–30 and 31–40 was 2.4% (3/126), 1.4% (2/143) and 0.8% (1/121), respectively (Table 3).

**Table 3:** Characteristics and outcomes in 391 hospitalized children with confirmed COVID-19 in six waves of SARS-CoV-2 in Babol district, northern Iran (From March 7, 2020 to March 20, 2022).

Characteristic	Total	Mean Ct of real time PCR*			*p*-value
		9–20	21–30	31–40	
Overall	391	126	143	121	–
Median age, yrs, (IQR)	3 (8)	2 (8)	3 (9)	3 (7)	0.424^a^
Mean ± SD age, yrs	5.2 ± 5.3	5.1 ± 5.3	5.7 ± 5.6	4.8 ± 5	0.312^b^
Age group, yrs					
<1	52 (13.3)	16 (12.7)	16 (11.2)	20 (16.5)	0.679^c^
1–5	191 (49)	59 (46.8)	73 (51)	59 (48.8)	
6–10	70 (17.9)	27 (21.4)	22 (15.4)	21 (17.4)	
11–18	77 (19.7)	24 (19)	32 (22.4)	21 (17.4)	
Gender					
Boy	210 (53.8)	72 (57.1)	74 (51.7)	64 (52.9)	0.654^d^
Girl	180 (46.2)	54 (42.9)	62 (48.3)	57 (47.1)	
Underlying diseases					
CVD^1^	16 (4.1)	7 (5.6)	7 (4.9)	2 (1.7)	0.253^d^
Diabetes	5 (1.3)	3 (2.4)	2 (1.4)	0 (0.0)	0.248^d^
KD^2^	11 (2.8)	3 (2.4)	5 (3.5)	3 (2.5)	0.828^d^
Hypertension	1 (0.2)	0 (0.0)	1 (0.7)	0 (0.0)	–
Malignancies	7 (1.8)	2 (1.6)	1 (0.7)	4 (3.3)	0.276^d^
BND^3^	37 (9.5)	10 (7.9)	14 (9.8)	13 (10.7)	0.744^d^
RD^4^	1 (0.3)	0 (0.0)	0 (0.0)	1 (0.8)	–
GID^5^	2 (0.5)	0 (0.0)	1 (0.7)	1 (0.8)	–
LD^6^	1 (0.3)	0 (0.0)	1 (0.7)	0 (0.0)	–
HBD^7^	5 (1.3)	2 (1.6)	2 (1.4)	1 (0.8)	0.858^d^
Others^8^	6 (1.5)	1 (0.8)	2 (1.4)	3 (2.5)	0.552^d^
Comorbidities					
No-Comorbidities	306 (78.5)	98 (77.8)	113 (79)	95 (78.5)	0.544^c^
One	76 (19.5)	27 (21.4)	25 (17.5)	24 (19.8)	
≥ Two	8 (2.1)	1 (0.8)	5 (3.5)	2 (1.7)	
Hospitalization outcome					
Length of stay, days, median (IQR)	4.5 (4)	4 (4)	4 (4)	6 (4)	0.001^a^
Length of stay, days, Mean ± SD	6.3 ± 6.6	5.5 ± 5.8	5.8 ± 5.2	7.9 ± 8.4	<0.001^b^
ICU admission	27 (6.9)	11 (8.7)	9 (6.3)	7 (5.8)	0.616^d^
In-hospital death	6 (1.5)**	3 (2.4)	2 (1.4)	1 (0.8)	0.602^d^

1. CVD: cardiovascular diseases; 2. KD: kidney diseases; 3. BND: brain & neurologic disorders; 4. RD: respiratory diseases; 5. GID: gastrointestinal diseases; 6. LD: liver diseases; 7. HBD: hematopoietic & blood disorders; 8. others: including diseases of immunodeficiency, Lupus, special diseases, and thyroiditis.

^a^ Median test was used.

^b^ Kruskal-Wallis test was used for mean age groups.

^c^ Cochrane-Armitage test for linear associations.

^d^ Chi-square, generalized Fisher exact tests were used to compare groups.

* The Ct value (Real time PCR) of one positive case was unknown.

** In one case, the Ct value of real time PCR positive was unknown.

### 3.4 Clinical outcomes

Over two years, out of 2,731 hospitalized patients with suspected COVID-19, 23 patients with a mean age of 6.4 ± 6.3 years (median: 4, IQR: 25) died (mortality rate: 0.8%), all during their hospital stay. On the other hand, 2,708 patients were discharged after treatment. Of the 23 patients who died during hospitalization, 7 (30.4%) were PCR-positive.

Most deaths among all suspected and RT-PCR-positive patients were in women (73.9% and 71.4%, respectively). Table 4 shows that most deaths, both overall and among rRT-PCR-positive patients, were in the 1–5 age group. BND was the most common underlying medical condition found in the overall group and in the group of RT-PCR-positive deaths (26.1% and 42.8% of deceased patients, respectively).

**Table 4:** Clinical and demographic characteristics of 2,731 hospitalized patients during a period when SARS-CoV-2 variant was prevalent in Babol district, northern Iran, among survivors and non-survivors overall and among real-time PCR-positive cases (From March 7, 2020 to March 20, 2022).

Variable	Total			Real-time PCR positive		
	Survivors (*N* = 2,708) *n* (%)	No survivors (*N* = 23) *n* (%)	*P* value	Survivors (*N* = 384) *n* (%)	No survivors (*N* = 7) *n* (%)	*P* value
Median age, yrs, (IQR)	3 (6)	4 (25)	0.479^a^	3 (8)	3 (11)	0.800^a^
Mean ± SD age, yrs	4.6 ± 4.5	6.4 ± 6.3	0.422^b^	5.2 ± 4.3	6 ± 6.5	0.861^b^
Age group, yrs						
<1	309 (11.4)	4 (17.4)	0.051^c^	51 (13.3)	1 (14.3)	0.940^c^
1–5	1,527 (56.4)	8 (34.8)		189 (49.2)	3 (42.9)	
6–10	510 (18.8)	4 (17.4)		69 (18)	1 (14.3)	
11–18	362 (13.4)	7 (30.4)		75 (19.5)	2 (28.6)	
Gender						
Boy	1,477 (54.6)	6 (26.1)	0.01^d^	208 (54.2)	2 (28.6)	0.257^d^
Girls	1,231 (45.5)	17 (73.9)		176 (45.8)	5 (71.4)	
Underlying Diseases						
CVD^1^	49 (1.8)	2 (8.7)	0.067^d^	15 (3.9)	2 (28.6)	0.002^d^
Diabetes	24 (0.9)	0 (0.0)	–	5 (1.3)	0 (0.0)	–
KD^2^	50 (1.8)	2 (8.7)	0.07^d^	11 (2.9)	0 (0.0)	–
Hypertension	6 (0.2)	0 (0.0)	–	1 (0.3)	0 (0.0)	–
Malignancies	69 (2.5)	3 (13)	0.021^d^	7 (1.8)	0 (0.0)	–
BND^3^	177 (6.5)	6 (26.1)	0.003^d^	34 (8.9)	3 (42.9)	0.002^d^
RD^4^	20 (0.7)	0 (0.0)	–	1 (0.3)	0 (0.0)	–
GID^5^	5 (0.2)	0 (0.0)	–	2 (0.5)	0 (0.0)	–
LD^6^	12 (0.4)	0 (0.0)	–	1 (0.3)	0 (0.0)	–
HBD^7^	57 (2.1)	1 (4.3)	0.391^d^	5 (1.3)	0 (0.0)	–
Special diseases	18 (0.6)	0 (0.0)	–	6 (1.6)	0 (0.0)	–
Others^8^	29 (1.1)	0 (0.0)	–	6 (1.6)	0 (0.0)	–
Comorbidity						
No-Comorbidity	2,260 (83.5)	10 (43.5)	<0.001^c^	304 (79.2)	2 (28.6)	0.002^c^
One	399 (14.7)	12 (52.2)		72 (18.8)	5 (71.4)	
Two ≤	49 (1.8)	1 (4.3)		8 (2.1)	0 (0.0)	
Disease Waves						
First	87 (3.2)	2 (8.7)	0.009^c^	3 (0.8)	0 (0.0)	0.866^c^
Second	268 (9.9)	2 (8.7)		69 (18)	1 (14.3)	
Third	303 (11.2)	1 (4.3)		29 (7.6)	1 (14.3)	
Fourth	181 (6.7)	4 (17.4)		18 (4.7)	0 (0.0)	
Fifth	513 (18.9)	9 (39.1)		118 (30.7)	3 (42.9)	
Sixth	347 (12.8)	2 (8.7)		82 (21.4)	2 (28.6)	
Null Wave^9^	1,009 (37.3)	3 (13)		65 (16.9)	0 (0.0)	
Hospitalization outcome						
Length of stay, days, median (IQR)	5 (4)	8 (30)	0.229^a^	4 (4)	14 (34)	0.057^a^
Length of stay, days, Mean ± SD	6.5 ± 6.1	14.9 ± 16.4	0.021^b^	6.1 ± 6	19.1 ± 17.4	0.015^b^
ICU admission	76 (2.8)	9 (39.1)	<0.001^d^	26 (6.8)	1 (14.3)	0.396^d^

1. CVD: cardiovascular diseases; 2. KD: kidney diseases; 3. BND: brain & neurologic disorders; 4. RD: respiratory diseases; 5. GID: gastrointestinal diseases; 6. LD: liver diseases; 7. HBD: hematopoietic & blood disorders; 8. others: including diseases of immunodeficiency, and thyroiditis; 9. Null wave: referring to the time between waves of SARS-CoV-2 infections (approximately 208 days).

^a^ Median test was used.

^b^ Mann–Whitney U test was used for mean age groups.

^c^ Cochrane-Armitage test for linear associations.

^d^ Chi-square, generalized Fisher exact tests were used to compare groups.

**Figure 1:**
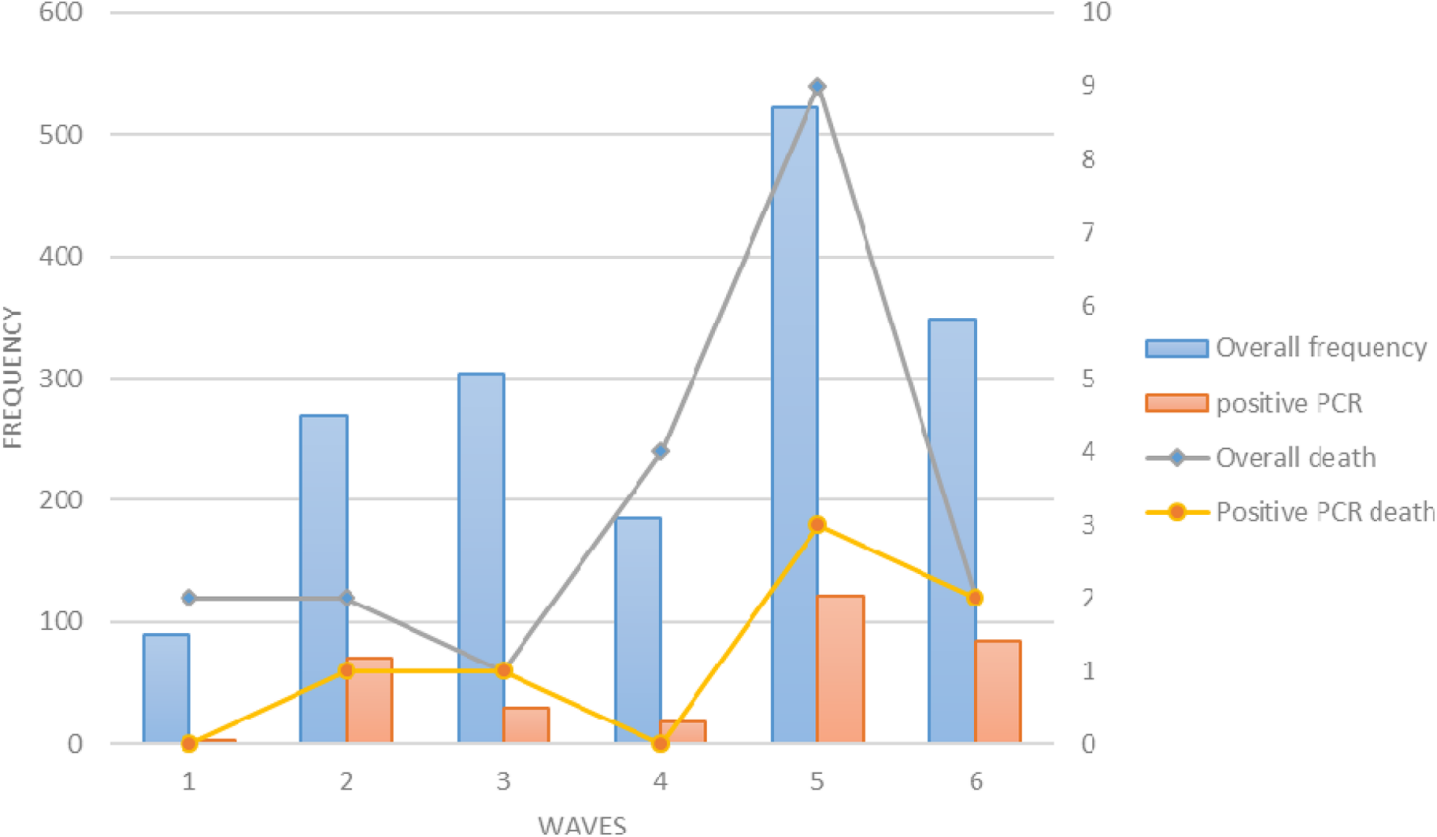
The frequency of hospitalizations (blue bars), positive PCR (orange bars), total deaths (black line), and deaths with positive PCR (yellow line) in children in six waves of COVID-19.

Of the patients who died, the highest proportion occurred in the fifth wave; 9 of 23 total deaths (39%) and 3 of 7 RT-PCR-positive deaths (42%) were attributed to the fifth wave. The lowest mortality rate in the overall suspected group was observed in the third wave with a percentage of 4.3%, and the lowest mortality rate in the RT-PCR-positive patients was observed in the first and fourth waves (Figure 1).

There was a significant difference in the duration of hospitalization between the deceased patients and the group of surviving patients. This difference was observed not only in the total suspected COVID-19 patients (2,731) but also in the laboratory-confirmed RT-PCR-positive patients (391). The mean duration of hospitalization in the 2,731 suspected patients was 14.9 ± 16.4 in the non-survivors compared to the survivors, which was 6.5 ± 6.1 (*P* = 0.021). In contrast, in the RT-PCR-positive group, the mean duration of hospitalization was 19.1 ± 17.4 for non-survivors and 6.1 ± 6 for survivors (*P* = 0.015) (Table 4).

### 3.5 Survival analysis

According to Table 5, the risk of death is twice as high in children with CVD and BND as in other children (HR = 2.54, *p*-value = 0.049, HR = 2.64, *p*-value = 0.189, respectively). The crude and adjusted hazard ratios are shown in Table 5.

**Table 5:** Survival analysis of confirmed COVID-19 hospitalized children in Babol district, northern Iran, during six waves (From March 7, 2020 to March 20, 2022).

Parameters	Crude			Adjusted		
	Hazard ratio	95% CI HR	*P-*value*	Hazard ratio	95% CI HR	*P-*value**
Age	0.991	0.88–1.12	0.888	1.014	0.84–1.22	0.884
Gender (girls)^1^	2.191	0.42–11.41	0.351	1.915	0.25–14.40	0.528
Mean Ct^2^						
9–20	9.190	0.83–101.35	0.070	4.958	0.33–75.09	0.248
21–30	4.072	0.36–45.77	0.255	1.865	0.11–32.069	0.668
Underlying diseases						
CVD^3^	6.445	1.01–41.11	0.049	2.546	0.17–37.04	0.494
BND^4^	2.928	0.59–14.56	0.189	2.645	0.29–23.82	0.386

1. Reference category: boys; 2. Reference category: real-time PCR 31–40; 3. CVD: cardiovascular diseases; and 4. BND: brain & neurologic disorders.

* *p*-value from univariate Cox regression.

** *p*-value from multivariable Cox regression, each variable was adjusted for the other variables listed in the table above.

## 4 Discussion

The present study examined the demographic, epidemiological and RT-PCR findings and outcomes of hospitalized COVID-19 patients <18 years old in a pediatric referral hospital affiliated with the Babol district in northern Iran during the two years of the pandemic and the six COVID-19 episodes. The current study included 2,731 hospitalized patients with suspected COVID-19 and 391 laboratory-confirmed SARS-CoV-2-positive hospitalized patients. The admission and mortality rates of the ICU population in the present study were 6.9% and 1.8%, respectively. The results of the current study showed that at least one comorbidity was associated with a higher mortality rate, with BND and CVD recorded as the two statistically significant comorbidities related to the higher mortality rate. There were no statistically significant differences in Ct values between these periods of SARS-CoV-2 infection.

The analysis of the present study differs from most recent studies, as this study analyzed information from six waves of the SARS-CoV-2 infection epidemic (23–26, 15). The time series data of the current study and the WHO European coronavirus statistics demonstrate an approximately similar distribution of patients over time (27).

The results of the current study, in which recorded RT-PCR laboratory-confirmed cases of COVID-19 were recorded in all age groups of children <18 years old, are consistent with those of Salvado et al. (28). They examined the epidemiologic and clinical characteristics of children with confirmed COVID-19 infection in a tertiary referral hospital in Manila, Philippines, and also showed that children of all age groups are susceptible to COVID-19 infection. Considering the results of these two studies, no specific age group <18 years old is immune to SARS-CoV-2 infection. The RT-PCR-positive results could not be recorded for the age group <1 and 6–10 years during the first wave, which may be due to the local transmission pattern and lower transmissibility of ancestral SARS-CoV-2 lineages and the lack of accurate diagnostic methods, especially at the beginning of the pandemic.

In the present study, no significant difference in terms of age and age classification was found between the six waves of SARS-CoV-2 infection. This is consistent with the findings of Salama et al. who evaluated the demographic and clinical characteristics of hospitalized individuals <18 years of age during exactly the same time period and duration and with a similar age classification as in our study (29). Additionally, the results of the ongoing study are similar to those of Alteri et al. who examined the epidemiologic characteristics and clinical presentation of SARS-CoV-2 in children during the pandemic waves (6). Although Alteri et al. and the current study found no significant difference between the different age groups (<1 year, 1–5 years, and >5 years) of clinically suspected and laboratory-confirmed RT-PCR-positive patients, the 1–5 age group was the most prevalent in both studies. The present study indicated the fifth wave, Delta variant, as the most prevalent for this particular age group, whereas Alteri et al. suggested the second wave as the most prevalent in this particular age group.

The present study demonstrated that the Delta variant of SARS-CoV-2, the fifth wave of SARS-CoV-2 infection, was associated with the highest number of hospitalized patients aged <18 years with clinical suspicion and laboratory confirmation. This result agrees with the result of a previous study by Takács et al. who declared that the age group <18 years was significantly susceptible to SARS-CoV-2 infection during the fifth wave (Delta variant B.1.617.2). Similar to the current study, they presented that most of the patients who tested positive for RT-PCR were documented during the Delta wave (30).

Regarding the Ct value determined in the current study, the following should be noted: Both the present study and the study by Alteri et al. (6) revealed that the mean Ct value varied significantly between the different waves of SARS-CoV-2, and in both studies, no differences were found between the different age groups for the mean Ct value in children <18 years of age. Although our results showed a statistically significant difference in Ct values between various waves of SARS-CoV-2 infection, no significant relationship was observed between mean Ct values and patient outcomes and disease severity, such as ICU admission or mortality rate.

These findings align with the study by Al-Shareef et al. (31), which failed to demonstrate a relationship between Ct levels and disease severity, particularly in the pediatric population.

In contrast, some studies reported an inverse correlation between Ct levels and clinical outcomes, severity and prognosis of SARS-CoV-2 infection, i. e. lower SARS-CoV-2 Ct levels lead to worse clinical outcomes, a more severe form of SARS-CoV-2 infection, a higher rate of hospitalization and a longer duration of hospitalization, and a higher likelihood of ICU admission with a longer ICU length of stay (32–34).

The findings of the ongoing study diverge from previous studies that found a correlation between Ct levels and clinical outcomes and prognosis of SARS-CoV-2 infection. This divergence could be due to the different demographic study populations, as these studies only included adults in their analyses.

Since our study showed a significant difference between Ct values and different waves of COVID-19, the fact that higher mean values of Ct in the initial phases of the COVID-19 pandemic, besides the lower cases of RT-PCR-positive patients in the current study, requires special attention (35). The fact that RT-PCR-positive patients with higher Ct levels, as representatives of the delayed phase with lower viral load, were predominantly found in the early phases of the COVID-19 pandemic, especially the first wave, may indicate that the patients in the early phases were mainly recognized by the delayed complications associated with SARS-CoV-2 such as multisystem inflammatory syndrome in children (MIS-C) rather than by the SARS-CoV-2 infection itself. These explanations are compatible with the reports of the study by Takács et al. (30), who identified the MIS-C cases in the early stages of the pandemic.

The current study illustrated that approximately 21% of RT-PCR-positive hospitalized patients and 71% of hospitalized children who died during observation had at least one comorbidity (statistically significant difference between with and without comorbidity with morality, *p*-value < 0.05). However, compared to the results of Salvado et al. (28), the present results suggested a lower percentage of patients who tested positive for RT-PCR and had at least one comorbidity (28) (21% vs. 48.7%). The percentage of patients who died during the observation and had at least one comorbidity was higher in the current study than in the study of Oliveira et al. (50.6% vs. 71%). Although the percentage of patients with at least one comorbidity was lower in our RT-PCR-positive patients than in the study by Salvado et al. (21% vs. 48.7%), a statistically significant association between at least one comorbidity and mortality was found in both studies (*p*-value < 0.05 in both studies).

The findings of the ongoing study indicated that BND was the most common underlying comorbidity in hospitalized patients aged <18 years, which is in line with the results of a previous study by Salama et al. (29). The current study revealed that BND was the predominant underlying disease during the Delta (fifth) and Omicron (sixth) waves. These results are consistent with those of Choi et al. (36), who examined the clinical characteristics of COVID-19-positive children during the Delta and Omicron waves.

The present study not only found that BND was the predominant underlying disease during the observation period in hospitalized children with SARS-CoV-2 but also that the presence of BND in these patients was associated with a higher mortality rate with a hazard ratio of 4.57 (95% CI: 1.56–13.34), which is with the same as the findings of Havers et al. (37), who claimed that children with neurological disorders had a 5–7 times higher risk of developing severe form of the disease.

Several studies found that BND increases the likelihood of getting a severe form of other viral infections related to the respiratory system, such as respiratory syncytial virus (RSV) or influenza (37–40). Furthermore, decreased muscle tone, reduced mobility, lower secretion output and higher risk of aspiration due to neurological disorders, as shown in a recent study (40), may contribute to a higher likelihood of lower respiratory tract infection, disease severity and mortality. This may explain the results of the present study, indicating that BND was significantly correlated with a higher mortality rate.

About 11% of the patients in the meta-analysis by Toba et al. (41) had to be treated in ICU. According to the current study, this percentage was slightly lower. Approximately 7% of laboratory-confirmed PCR-positive hospitalized children in the present study were admitted to the ICU. The ongoing study had the highest number of hospitalized cases and the highest ICU admission rate during the Delta wave. These findings are consistent with the results of Jelic et al. (42), who observed a similar trend in a study of the characteristics of children with COVID-19 in Colorado hospitalized during different variants. This illustrated that the Delta wave was associated with a greater number of hospitalizations and more severe cases of infection that required more ICU admissions than other waves.

The current study has certain limitations, such as the difficulty in interpreting clinical symptoms, the lack of CT scans for the participants and the possibility of errors during the pre-test phase. Moreover, data on previous SARS-CoV-2 infections and complications after COVID-19 were not accessible. Furthermore, the data on patients' comorbidities was based on self-reporting by the subjects. In addition, in the ongoing study, the direct impact of the use of COVID-19 medications (antivirals, monoclonal antibodies, and immunotherapy) on disease severity could not be assessed as no information on the patients was available. These factors could explain the differences in outcomes observed during the different COVID-19 waves after the general introduction of vaccination in Iran.

## 5 Conclusion

In conclusion, this study sheds light on the critical aspects of the impact of the SARS-CoV-2 pandemic on the <18-year-old population in Iran. By carefully observing six waves of illness over a two-year period, the study provides invaluable insights into the demographic, clinical and outcome waves of COVID-19 cases among pediatric patients. The study investigated confirmed suspected clinical cases and provided new insights into previously unclear areas. In particular, ICU admission and mortality rates were carefully documented, with an admission rate of 6.9% and a mortality rate of 1.8%.

The study results emphasize that children of all ages can be infected with SARS-CoV-2, which is consistent with previous research. The present study has shown that individuals aged 1–5 years have a higher risk of contracting COVID-19. However, it is essential to note that no particular age group can be considered completely immune to the virus. The results indicate how important comorbidities are for the disease, with BND likely to be a significant factor associated with higher mortality.

## Data Availability

The original contributions presented in the study are included in the article/Supplementary Material, further inquiries can be directed to the corresponding author.
